# The Protective Effects of Melatonin Against LPS-Induced Septic Myocardial Injury: A Potential Role of AMPK-Mediated Autophagy

**DOI:** 10.3389/fendo.2020.00162

**Published:** 2020-04-16

**Authors:** Shouyin Di, Zheng Wang, Wei Hu, Xiaolong Yan, Zhiqiang Ma, Xiaofei Li, Weimiao Li, Jianyuan Gao

**Affiliations:** ^1^Department of Geriatrics, Xijing Hospital, The Fourth Military Medical University, Xi'an, China; ^2^Department of Thoracic Surgery, Sixth Medical Center of PLA General Hospital, Beijing, China; ^3^Department of Thoracic Surgery, Tangdu Hospital, The Fourth Military Medical University, Xi'an, China; ^4^Department of Cardiothoracic Surgery, Central Theater Command General Hospital of Chinese People's Liberation Army, Wuhan, China; ^5^Department of Immunology, The Fourth Military Medical University, Xi'an, China; ^6^Department of Oncology, the Second Affiliated Hospital of Xi'an Jiaotong University, Xi'an, China

**Keywords:** melatonin, lipopolysaccharide, septic myocardial injury, AMP-activated protein kinase, autophagy

## Abstract

**Aim:** Melatonin is an indolamine secreted by the pineal gland, as well as most of the organs and tissues. In addition to regulating circadian biology, studies have confirmed the multiple pharmacological effects of melatonin. Melatonin provides a strong defense against septic myocardial injury. However, the underlying mechanism has not been fully described. In this study, we investigated the protective effects of melatonin against lipopolysaccharide (LPS)-induced myocardial injury as well as the mechanisms involved.

**Methods:** Mice were intraperitoneally injected with LPS to induce a septic myocardial injury model or an LPS shock model, depending on the dose of LPS. Melatonin was given (20 mg/kg/day, via intraperitoneal injection) for a week prior to LPS insult. 6 h after LPS injection, echocardiographic analysis, TUNEL staining, transmission electron microscopy (TEM), western blot, quantitative real-time PCR and ELISA were used to investigate the protective effects of melatonin against LPS induced myocardial injury. AMPK inhibitor, autophagy activator and inhibitor, siRNAs were used for further validation.

**Results:** Survival test showed that melatonin significantly increased the survival rate after LPS-induced shock. In the sepsis model, melatonin markedly ameliorated myocardial dysfunction, decreased the release of inflammatory cytokines, activated AMP-activated protein kinase (AMPK), improved mitochondrial function, and activated autophagy. To confirm whether the protection of melatonin was mediated by AMPK and autophagy, Compound C, an AMPK inhibitor; 3-MA, an autophagy inhibitor; and Rapamycin (Rapa), an autophagy activator, were used in this study. AMPK inhibition down-regulated autophagy, abolished protection of melatonin, as indicated by significantly decreased cardiac function, increased inflammation and damaged mitochondrial function. Furthermore, autophagy inhibition by 3-MA significantly impaired the protective effects of melatonin, whereas autophagy activation by Rapa reversed LPS + Compound C induced myocardial injury. In addition, *in vitro* studies further confirmed the protection of melatonin against LPS-induced myocardial injury and the mechanisms involving AMPK-mediated autophagy signaling.

**Conclusions:** In summary, our results demonstrated that melatonin protects against LPS-induced septic myocardial injury by activating AMPK mediated autophagy pathway.

## Introduction

Sepsis is a whole-body inflammatory response caused by infection, and it has high incidence and mortality rates (1, 2). The mortality rate of sepsis shock reaches 70% or even higher (1, 3). Lipopolysaccharide (LPS), also known as lipoglycans or endotoxin, is the major pro-inflammatory component of Gram-negative bacteria. It has been reported that LPS is one of the main triggers for sepsis and septic organ dysfunction (4, 5). Notably, myocardial injury is one of the most important characteristics of sepsis; it significantly increases the mortality rate of sepsis (6–8). Some studies have demonstrated that LPS-induced acute myocardial injury is mainly caused by several pro-inflammatory cytokines, such as tumor necrosis factor-α (TNF-α), interleukin 1β (IL-1β) and IL-6 (5, 9, 10).

Autophagy is an important cellular process that disassembles damaged organelles in the cell. Interestingly, decreased autophagy was reported to contribute to the pathogenesis and severity of sepsis, especially septic myocardial injury (11–13). The mechanisms involved can be attributed to the increased secretion of pro-inflammatory cytokines (14–16). These imply the potential role of autophagy in decreasing pro-inflammatory cytokines and protecting against septic injury. Notably, LPS-induced acute myocardial injury can be significantly attenuated by upregulation of autophagy (17, 18). The activation of AMP-activated protein kinase (AMPK) signaling is effective in upregulating autophagy (19, 20). It might be a promising way to alleviate LPS-induced acute myocardial injury via the activation of AMPK signaling, upregulation of autophagy, and suppression of inflammation.

Melatonin, an endogenous hormone secreted by the pineal gland, as well as most of the organs and tissues of the organisms, has pleiotropic biological and pharmacological properties, such as anti-oxidative stress (21), anti-inflammation (22, 23), anti-fibrosis (24), anti-carcinogenesis (25, 26), and maintaining cellular homeostasis (27). Our previous studies demonstrated that melatonin can protect the heart against ischemia-reperfusion injury (28, 29). Other studies also support the protective effects of melatonin against heart failure (30–32) and drug-induced myocardial injury (33). Notably, melatonin has been shown to protect against LPS-induced mitochondrial impairment and myocardial injury (34, 35). However, the exact underlying mechanism is still unclear. Additionally, melatonin is an autophagy regulator (36, 37) and an activator of AMPK signaling (10). These evidences suggest a potential role of AMPK and autophagy in melatonin induced cardiac protection. However, the exact mechnisam remains unclear.

Based on previous studies, we hypothesized that melatonin might induce cardiac protection via activating AMPK rugulated autophagy, and further inhibiting mitochondrial injury and myocardial apoptosis. The aim of our study was to analyse in depth the molecular mechanisms involved in the melatonin cardioprotection against LPS-induced injury. We investigated the protective effects of melatonin against acute myocardial injury induced by LPS in mice, and we further investigated the possible mechanisms *in vivo* and *in vitro*. Firstly, we established an LPS-induced myocardial injury model on C57BL/6J mice, and observed the beneficial effects of melatonin on survival rate and cardiac function. Then, we investigated the potential mechanisms involved in this process, including AMPK and autophagy pathway. Furthermore, AMPK and autophagy inhibitor were used to block the protection of melatonin to confirm that AMPK and autophagy activation play a key role in melatonin induced myocardial protection. Then autophagy activator was used to confirm that AMPK exerts myocardial protection is autophagy dependent. In the end, primary neonatal rat cardiomyocytes were used to further verify the mechanisms found *in vivo*. The results in this study provide a promising way to protect against sepsis induced myocardial damage.

## Materials and Methods

### Materials

LPS from *Escherichia coli* serotype O55:B5, Compound C, Rapamycin (Rapa) and 3-Methyladenine (3-MA) were purchased from Sigma-Aldrich (St. Louis, MO, USA). Antibodies against phosphorylated nuclear factor kappa-B (pNF-κB), inhibitor of NF-κB α (IκBα), pAMPK (Thr 172), AMPK, phosphorylated mammalian target of rapamycin (pmTOR), mTOR, p62, light chain 3B (LC3B), B-cell lymphoma 2 (Bcl-2), Bcl-2-associated X protein (Bax) were purchased from Cell Signaling Technology (Beverly, MA, USA). Antibody against Tubulin was purchased from CMCTAG (Milwaukee, WI, USA). Goat-anti-mouse IgG and goat-anti-rabbit IgG were purchased from Zhongshan Company (Beijing, China). Mouse IL-6 ELISA kit and Mouse TNF-α ELISA kit were purchased from Elabscience biotechnology (Wuhan, Hubei, China). TRIzol total RNA extraction kit was purchased from Tiangen Biotech (Beijing, China). PrimeScript RT Master Mix, SYBR Premix Ex Taq II and primers were purchased from TAKARA (Dalian, Liaoning, China). siRNAs were designed and synthesized by GenePharma (Shanghai, China). Lipofectamine 3000 was purchased from Invitrogen (Carlsbad, CA, USA). Mitochondrial extraction kit was purchased from Beyotime Biotechnology (Shanghai, China). Glutathione peroxidase (GPx) assay kit (Colorimetric method), GSH reductase (GRd) assay kit, reduced GSH assay kit (Spectrophotometric method), total GSH assay kit, electron transport chain Complex I assay kit and electron transport chain Complex II assay kit were purchased from Nanjing Jiancheng Bioengineering Institute (Nanjing, Jiangsu, China). Fetal bovine serum and BCA protein assay kit were purchased from ThermoFisher Scientific (Waltham, MA, USA). Terminal deoxynucleotidyl transferase dUTP nick end labeling (TUNEL) kits were purchased from Roche (Mannheim, Germany).

### Animals and Treatment

Male C57BL/6 mice weighing 20 g to 22 g (10–12-week-old) were purchased from Laboratory Animal Center of Fourth Military Medical University. All experiments were performed in adherence to the National Institutes of Health Guidelines for the Use of Laboratory Animals. The study protocols were approved by the Fourth Military Medical University Committee on Animal Care. The mice had free access to food and water and were bred at 26°C with a 12 light /12 h dark cycle.

The septic myocardial injury model was established using a 6 mg/kg LPS intraperitoneal injection. 6 h after LPS injection, cardiac function was detected. Hearts of mice in each group were collected immediately for further assays. In survival test, 30 mg/kg LPS was given intraperitoneally (38). These mice were kept and monitored for lethality every 6 h for 3 days (*n* = 15).

There were four parts in the *in vivo* study (Table 1). In the first part (test of survival), mice were divided into 3 groups, namely control group, LPS group and LPS + melatonin group (*n* = 15). In the second part, mice were divided into control group, melatonin group, LPS group and LPS + melatonin group (20 mice in each group). In the third part, mice were divided into LPS group, LPS + Compound C group, LPS + melatonin group, and LPS + melatonin + Compound C group (20 mice in each group). In the fourth part, mice were divided into LPS + melatonin group, LPS + melatonin + 3-MA group, LPS + Compound C group, and LPS + Compound C + Rapamycin group (20 mice in each group).

**Table 1:** Experimental schedule and group assignment.

	**Group assignation**	**Animal number**	**Injections and dose**	**Time of sacrifice/** **tissue collection**
Figure 1A	Control	15	LPS: 30 mg/kg, once, intraperitoneally Mel: 20 mg/kg/day, for 7 days before LPS treatment, intraperitoneally	-
	LPS			
	LPS+Mel			
Figures 1–3	Con	20	LPS: 6 mg/kg, once, intraperitoneally Mel: 20 mg/kg/day, for 7 days before LPS treatment, intraperitoneally	6 h after LPS treatment
	Mle			
	LPS			
	LPS+Mel			
Figures 4–6	LPS	20	LPS: 6 mg/kg, once, intraperitoneally Mel: 20 mg/kg/day, for 7 days before LPS treatment, intraperitoneally Compound C: 20 mg/kg, once, 30 min prior to the LPS injection	6 h after LPS treatment
	LPS+Compound C			
	LPS+Mel			
	LPS+Mel+Compound C			
Figures 7–9	LPS+Mel	20	LPS: 6 mg/kg, once, intraperitoneally Mel: 20 mg/kg/day, for 7 days before LPS treatment, intraperitoneally Compound C: 20 mg/kg, once, 30 min prior to LPS injection, intraperitoneally 3-MA: 10 mg/kg/day, for 7 days before LPS treatment, intraperitoneally Rapamycin: 1.5 mg/kg/day, for 7 days before LPS treatment, intraperitoneally	6 h after LPS treatment
	LPS+Mel+3-MA			
	LPS+Compound C			
	LPS+Compound C+Rapa			
Figure 10	Control	-	LPS: 5 μg/ml, for 24 h Mel: 100 μmol/l, 24 h before LPS treatment Compound C: 10 μmol/l, 1 h prior to LPS treatment	24 h after LPS was added
	LPS			
	LPS+Mel			
	LPS+Mel+Compound C			

Melatonin was given 20 mg/kg/day via an intraperitoneal injection for a week prior to the LPS insult. The dose and usage of melatonin were determined by our previous studies (39). As an activity inhibitor of AMPK, Compound C was intraperitoneally administrated at a dose of 20 mg/kg, 30 min prior to the LPS injection (40). Rapa, an autophagy activator, was given at 1.5 mg/kg intraperitoneally daily for up to 1 week before the LPS insult. 3-MA serves as an autophagy inhibitor, and it was given 10 mg/kg intraperitoneally daily for up to 1 week before the LPS insult (41). These reagents have no influence on mice survival. All the treatments were given about one hour after light onset in the morning.

### Cell Culture

Primary neonatal rat cardiomyocytes were prepared as previously described (42). Briefly, hearts from neonatal rats were taken, and the ventricles were cut into pieces and then digested with collagenase type II (1 mg/ml) and trypsin (4 mg/ml) in D-Hanks balanced salt solution (NaCl 8.00 g/L, NaHCO_3_ 0.35 g/L, Na_2_HPO_4_·12H_2_O 0.37 g/L, KCl 0.40 g/L and KH_2_PO_4_ 0.06 g/L). About 15 min later, cells were centrifuged, resuspended in cell culture flasks (90% DMEM/F-12 1:1 medium, 10% FBS, 100 U/ml of penicillin and streptomycin) and incubated at 37°C in a 5% CO_2_/95% air humidified incubator. After 90 min, non-cardiomyocytes were attached to the bottom of the flask while most of the cardiomyocytes were suspended in culture medium. The cardiomyocytes in the supernatant were collected by centrifugation, washed with phosphate buffer solution (PBS) and plated in new flasks.

### Cell Treatment and siRNA Transfection

An *in vitro* study was established to confirm the anti-inflammatory effect of melatonin and the underlying mechanisms, which have been found in *in vivo* studies. In the first part of the *in vitro* study, primary neonatal rat cardiomyocytes were divided into four groups, namely control group, LPS group, LPS + melatonin group, and LPS + melatonin + Compound C group. Cells were treated with 5 μg/ml LPS for 24 h (43). 100 μM melatonin was given 24 h prior to LPS insult). 10 μM Compound C was given 1 h prior to LPS insult (44, 45). In the second part of *in vitro* study, cells were subjected to ATG5 or mTOR siRNA, and further treated by LPS and melatonin. Cells were divided into 3 groups, control group, LPS group and LPS + melatonin group.

Specific RNA interference was performed utilizing ATG5 and mTOR siRNA, with a non-specific scramble siRNA as control. For transfection, cardiomyocytes were seeded in six-well plates, and siRNAs (90 pmol/L) were transfected using Lipofectamine 3000 and incubated in complete medium without antibiotics for 90 min. Then cells were washed and incubated for 24 h. mRNA levels and protein expressions were detected to confirm siRNA efficiency before usage.

### Echocardiographic Analysis

Echocardiographic analysis was established 6 h after LPS injection using the Esaote Twice System with the SL3116 transducer (Esaote, Genova, Italy). Two-dimensional and M-mode echocardiographic measurements were performed with a VEVO 2100 high-resolution *in vivo* imaging system. Left ventricular internal diameter (LVID);d, LVID;s, left ventricular ejection fraction (LVEF) and left ventricular fractional shortening (LVFS) were quantified based on the M-mode images. Isoflurane was used to anesthetize the mice, and the heart rates were maintained at 400–500 beats per min during the detection procedure.

### TUNEL Staining

Six hours after LPS insult, mice were sacrificed and mice hearts were collected. Then the hearts were stored in 4% paraformaldehyde for at least 24 h. After that, mice hearts were further embedded in paraffin and sectioned according to routine procedures. The sections were then deparaffinized with xylene and ethanol (100%, 95%, 90%, 80%, and 70%) and rinsed in PBS for 5 min. The sections were then subjected to TUNEL staining. TUNEL staining was performed under strict adherence to the manufacturer's instructions. Briefly, the paraffin sections were treated with 0.1% triton X-100 for 20 min at room temperature. The sections were then washed and exposed to the TUNEL reaction solution at 37°C for 60 min. Subsequently, the sections were washed with PBS and incubated with DAPI at 37°C for 15 min to identify the cell nucleus. Finally, the sections were washed with PBS, covered with a coverslip and examined with an Olympus FV1000 laser scanning confocal microscope (Olympus, Japan). The apoptotic cells were visually identified in 10 randomly selected areas and photographed at a magnification of ×200.

### Extraction of Mitochondria

A tissue mitochondria isolation kit was used in this experiment. Briefly, 6 h after LPS insult, mice were sacrificed and the hearts were collected. Then the mice hearts were minced in PBS and ice-bathed for 10 min. Then, the tissue was centrifuged at 600 g for 20 s (4°C). The pellet fractions were collected, and 1 ml trypsin was added for 20 min. Then, the tissue was centrifuged at 600 g for 20 s (4°C), and the pellet fraction was washed with buffer A and centrifuged at 600 g for 20 s (4°C). The pellet fractions were then homogenized in 1 ml of Buffer A and centrifuged at 1,000 g for 5 min (4°C). Then, the supernatant fraction was centrifuged at 11,000 g for 10 min (4°C). The final pellet fraction was the mitochondria.

### Detection of Complex I and Complex II Activity

Complex I (NADH-CoQ reductase) and complex II (succinate dehydrogenase) activities were measured spectrophotometrically with a plate reader using standard coupled enzyme assays as previously described (46, 47). Activities were expressed in mU/min/mg of wet muscle weight.

### Measurement of GPx and GRd Activities and Glutathione (GSH) and Glutathione Disulfide (GSSG) Levels

GPx and GRd activities were detected using a GSH-PX assay kit and a GSH reductase assay kit respectively. GSH levels were detected utilizing a reduced GSH assay kit (Spectrophotometric method), and GSSG levels were detected utilizing an oxidized GSH assay kit respectively. 6 h after LPS insult, the mice hearts were collected and perfused with saline for further detection. For GPx detection, mice hearts were homogenized and centrifuged, and 0.2 ml supernatant fraction was mixed with 0.2 ml GSH solution (1 mM) and left to react at 37°C for 5 min. Then, 0.1 ml buffer 1 working solution was added and reacted for 5 min. After that, 2 ml buffer 2 was added, and the mixture was centrifuged at 3,000 g for 10 min. Then, 1 ml supernatant fraction was taken and mixed with buffer 3, buffer 4, and buffer 5 for optical density measurement. Finally, GPx activity was calculated according to the manufacturer instructions. GRd activity detection was carried out according to the manufacturer instructions. Briefly, 20 μl supernatant fraction was added to 2.4 ml working solution, and the OD was detected 30 s and 2 min later, respectively. GRd activity was calculated according to the instructions. GPx is expressed as U/mg protein, and GRd activity is expressed as U/g protein.

GSH and GSSG levels were detected and calculated according to manufacturers' instructions.

### Detection of Serum Pro-inflammatory Cytokines via ELISA

Mice were euthanized 6 h after LPS injection. Then the blood was collected, rest for 15 min, and the serum was separated by centrifugation at 3,000 rpm for 20 min at 4°C. Isolated serum was stored at −80°C. IL-6 and TNF-α levels in serum were detected utilizing ELISA kits according to the manufacturer's instructions.

### Quantitative Real-Time PCR

Real-time PCR analysis was established as previously described (42). Briefly, after LPS treatment for 24 h, cells were collected and resuspended with TRIzol. Then total RNA was extracted from cells using the TRIzol total RNA extraction kit, and reverse transcription was performed using the Prime Script RT Master Mix. Then TNF-α and IL-6 mRNA levels were detected using quantitative real-time reverse transcriptase PCR analyses with SYBR Premix Ex Taq. The following primers were used. TNF-α forward primer: 5′-CCAATCTGTGTCCTTCTAAC-3′ and TNF-α reverse primer: 5′-GTTTCTGAGCATCGTAGTTG-3′. IL-6 forward primer: 5′-AAGGTCACTATGAGGTCTAC-3′ and IL-6 reverse primer 5′-CATATTGCCAGTTCTTCGTA-3′. β-actin forward primer: 5′- GGTCATCACTATCGGCAATG-3′ and β-actin reverse primer: 5′- AGGTCTTTACGGATGTCAAC-3′. The reaction conditions were as follows: [1] 95°C for 30 s, [2] 39 cycles of 95°C for 5 s, 60°C for 30 s, and 72°C for 30 s, [3] 72°C for 5 min. The expression levels of the examined transcripts were compared to that of β-actin and normalized to the mean value of the controls.

### Western Blot

Protein expressions were determined through western blot analysis as previously described (42). Heart tissue was harvested 6 h after LPS injection and then lysed in RIPA buffer for 20 min at 4°C. Lysates were centrifuged at 12,000 rpm for 20 min at 4°C. Total protein concentrations were detected using a BCA protein assay kit, and 20 μg total protein was resolved by SDS-PAGE. Separated proteins were transferred to PVDF membranes. Membranes were blocked with 5% defatted milk and incubated with antibodies against pNF-κB, IκBα, pAMPK, pmTOR, p62, LC3B, Bcl-2, Bax (1:1000), Tubulin (1:2500), β-actin (1:2000) and secondary antibodies. The fluorescent signal was detected using a BioRad imaging system (BioRad, Hercules, CA, USA), and the signal was quantified using Image Lab Software (BioRad, Hercules, CA, USA).

### Transmission Electron Microscopy (TEM)

Six hours after LPS injection, the hearts of mice were harvested and rinsed in PBS. The tissue was fixed in glutaraldehyde (2.5%) for at least 24 h. After that, samples were cut into 1-mm cubes and further fixed in 1% osmium tetroxide in 0.1 M potassium cacodylate buffer (pH 7.4) for 1 h at 4°C. Then cubes were dehydrated and embedded in Epon/SPURR resin. Sections of 75 nm thickness were stained with uranium acetate and lead citrate by a professional technician. Images were observed and captured utilizing a TECNAI G2 Spirit Biotwin TEM at 120 KV.

### Statistical Analysis

All of the values are presented as the means ± standard error of mean (SEM). Statistical significance (*P* < 0.05) for each variable was evaluated by one-way ANOVA. Survival curves (Kaplan-Meier curve) were analyzed using the log-rank test (GraphPad Software Inc., San Diego, CA, USA).

## Results

### Effect of Melatonin Treatment on Mice Survival After LPS Shock

**Figure 1:**
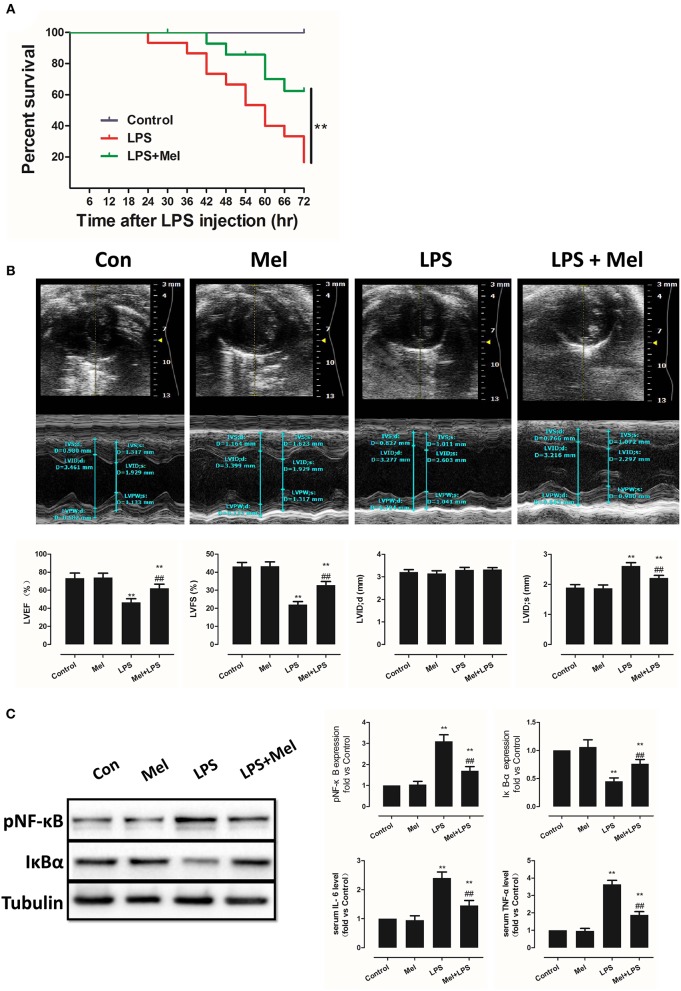
Effects of melatonin administration on survival, cardiac dysfunction and inflammation after LPS insult. **(A)** 30 mg/kg LPS was given to mice to assess the effects of LPS on survival rate. The mice survival rate was evaluated using Kaplan-Meier curves (*n* = 15). **(B)** Representative M-mode echocardiograms from mice in each group and quantitative analysis of the LVEF, FS, LVID; d, LVID; s. **(C)** Representative bands of pNF-κB, IκBα and Tubulin, quantitative analysis of pNF-κB, IκBα expression and serum IL-6, TNF-α levels. *n* = 6 **P < 0.05 vs. the control group, ^##^*P* < 0.05 vs. the LPS group. Mel, melatonin.

A higher dose of LPS (30 mg/kg) was used to induce LPS shock and to examine the protective effects of melatonin on mice survival. The mice were divided into 3 groups with 15 mice in each group, namely, a control group, LPS group and LPS + melatonin group. The treatment of each group has been described in the Methods section. The mice survival rate was evaluated using the Kaplan-Meier curve (Figure 1A). LPS significantly reduced mice survival rate (16.667%, *P* < 0.05 vs. control group). Melatonin treatment induced a significant increase in survival rate (62.338%, *P* < 0.05 vs. LPS group).

### Effects of Melatonin Treatment on Mice LPS-Induced Cardiac Dysfunction and Inflammation

A lower dose of LPS (6 mg/kg) was used to examine the role of melatonin on cardiac dysfunction and inflammation. Mice were divided into 4 groups, namely, a control group, melatonin group, LPS group and LPS + melatonin group. After LPS injection, echocardiography and hemodynamic measurements revealed decreased LVEF, LVFS, and increased LVID; s compared to the control group (Figure 1B, *P* < 0.05). Pre-treatment of melatonin reversed cardiac dysfunction induced by LPS as indicated by a higher LVEF, LVFS and a smaller LVID; s (Figure 1B, *P* < 0.05).

Furthermore, after LPS insult, IL-6 and TNF-α levels in serum and pNF-κB expression in heart tissue were increased (*P* < 0.05), and IκBα expression was decreased (*P* < 0.05). These effects were attenuated by melatonin administration (Figure 1C, *P* < 0.05).

### Effects of Melatonin on Apoptosis, AMPK and Autophagy Pathway

**Figure 2:**
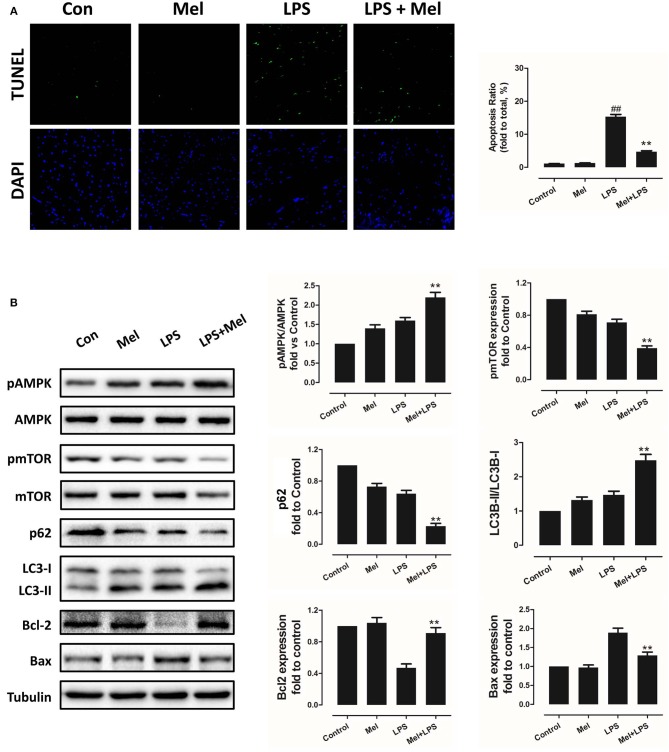
Effects of melatonin administration on myocardial apoptosis, AMPK and autophagy pathway after LPS injury. **(A)** Representative images of TUNEL staining and quantitative analysis of the apoptosis ratio. **(B)** Representative bands of western blot and quantitative analysis of pAMPK phosphorylation level, pmTOR phosphorylation level, p62 expression, LC3B-II/LC3B-I ratio, Bcl-2 expression and Bax expression. *n* = 6, **P < 0.05 vs. the LPS group, ^*##*^*P* < 0.05 vs. the Control group. Mel, melatonin.

Melatonin exerts cell protection via multiple pathways, including the AMPK pathway and the autophagy pathway. Hence, AMPK phosphorylation and autophagy activation were assessed, as well as myocardial apoptosis. Melatonin administration significantly abolished LPS-induced apoptosis (Figure 2A, *P* < 0.05). Western blot analysis of Bcl-2 and Bax further confirmed this effect (Figure 2B, *P* < 0.05). Interestingly, LPS insult slightly increased pAMPK and LC3B-II expression and decreased pmTOR and p62 levels, which means that AMPK and autophagy might be slightly activated. Melatonin treatment further significantly increased pAMPK and LC3B-II expression and decreased pmTOR and p62 levels (Figure 2B, *P* < 0.05).

**Figure 3:**
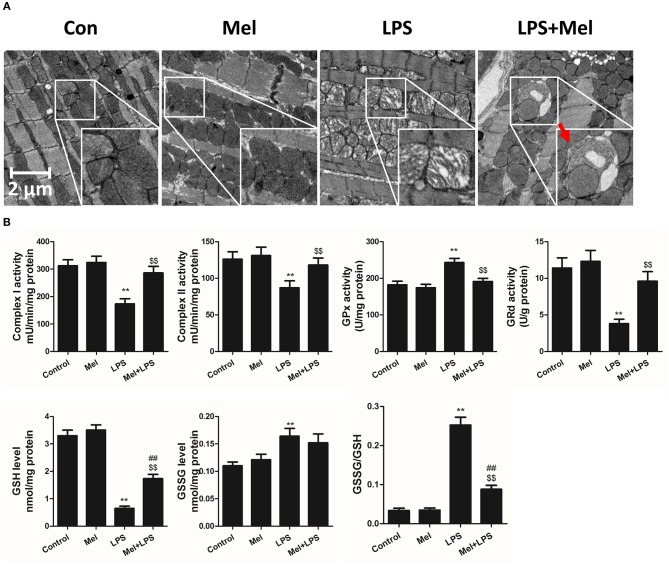
Effects of melatonin administration on mitochondrial ultrastructure and function after LPS injury. **(A)** Representative images of mitochondrial morphology. The red arrow indicates an autophagosome. **(B)** Quantitative analysis of mitochondrial Complex I, Complex II, GPx, and GPd activity, and GSH, GSSG levels. *n* = 6 **P < 0.05 vs. the control group, ^##^*P* < 0.05 vs. the Mel group, ^$$^*P* < 0.05 vs. the LPS group. Mel, melatonin.

Furthermore, TEM was carried out to detect the micro-histopathology of mice heart tissue. After LPS treatment, mitochondria swelling and local myofilament dissolving can be observed. Melatonin treatment reversed this condition and maintained an almost normal mitochondrial morphology. Moreover, autophagosomes can be observed in the melatonin-treated mice hearts, indicating activation of autophagy (Figure 3A). Furthermore, mitochondria function was detected to confirm the morphological changes. LPS treatment decreased Complex I, Complex II and GRd activity and GSH levels and increased GPx activity and GSSG levels (Figure 3B, *P* < 0.05). Melatonin treatment abolished this effect by increasing Complex I activity, Complex II activity, GRd activity and GSH levels and by decreasing GPx activity and GSSG levels (Figure 3B, *P* < 0.05).

### Inhibition of AMPK Abolished the Protection of Melatonin Against LPS Insult and Inhibited Autophagy Levels

**Figure 4:**
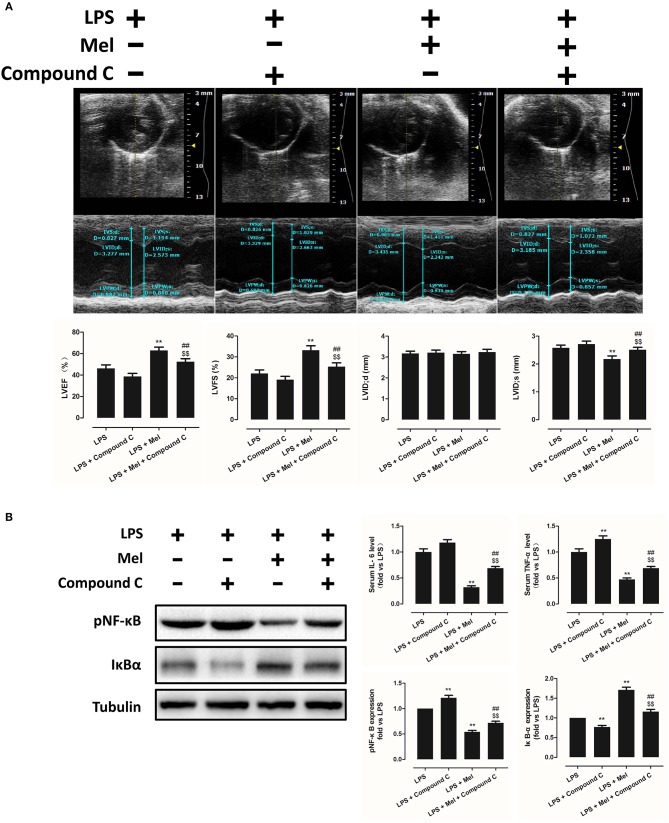
Compound C treatment exacerbated LPS-induced injury and impaired melatonin-induced cardiac protection. **(A)** Representative M-mode echocardiograms from mice in each group and quantitative analysis of mice LVEF, FS, LVID; d, LVID; s. **(B)** Representative bands of pNF-κB and IκBα, and quantitative analysis of pNF-κB, IκBα expressions and serum IL-6, TNF-α levels. *n* = 6. **P < 0.05 vs. the LPS group, ^##^*P* < 0.05 vs. the LPS + Compound C group, ^*$$*^*P* < 0.05 vs. the LPS + melatonin group. Mel, melatonin.

**Figure 5:**
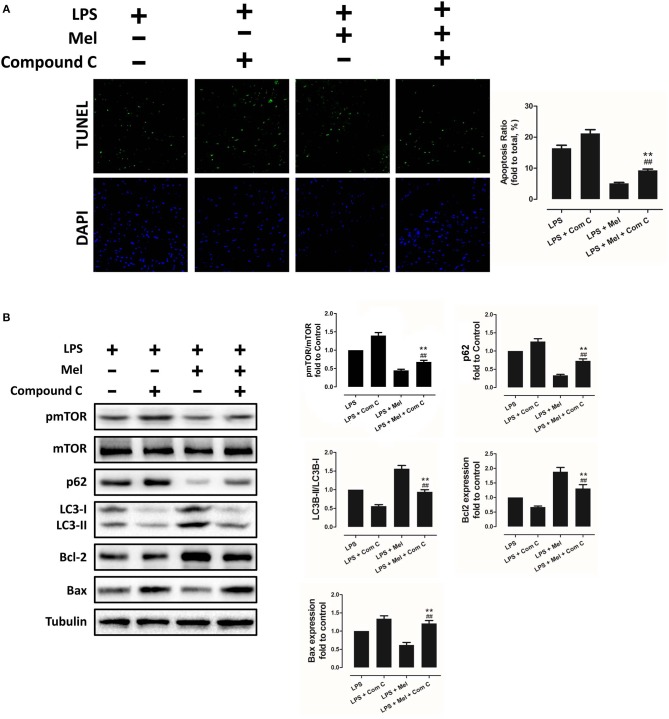
Compound C reversed melatonin-induced cardiac protection via inhibiting autophagy activation and increasing apoptosis. **(A)** Representative images of TUNEL staining and quantitative analysis of the apoptosis ratio. **(B)** Representative bands of western blot and quantitative analysis of pmTOR phosphorylation level, p62 expression, LC3B-II/LC3B-I ratio, Bcl-2 expression, and Bax expression. *n* = 6, **P < 0.05 vs. the LPS + Compound C group, ^##^*P* < 0.05 vs. the LPS + melatonin group. Mel, melatonin.

To further confirm the role of AMPK in melatonin-mediated myocardial protection, Compound C, a selective AMPK inhibitor, was used to inhibit AMPK activity. Compared to the LPS group, administration of LPS + Compound C exerted no significant influence on cardiac function, as indicated by LVEF and LVFS (Figure 4A). However, compared to the LPS group, further inhibition of AMPK led to an increase in serum IL-6 and TNF-α levels, as well as myocardial pNF-κB expression, and led to a decrease in IκBα expression (Figure 4B, *P* < 0.05). Furthermore, western blot analysis showed that Compound C increased pmTOR, p62 expression, reduced LC3B-II expression (Figure 5B, *P* < 0.05) and increased the Bax/Bcl-2 ratio (Figure 5B, *P* < 0.05). TUNEL staining also showed that Compound C administration increased myocardial apoptosis (Figure 5A, *P* < 0.05). These data indicated that inhibiting AMPK activity further increased the level of inflammation and abolished LPS-induced cell autophagy.

Compared to the LPS + melatonin group, LPS + melatonin + Compound C treatment decreased LVEF and LVFS (Figure 4A, *P* < 0.05); increased serum IL-6 levels, TNF-α levels and myocardial pNF-κB expression; and decreased IκBα expression (Figure 4B, *P* < 0.05). pmTOR and p62 expressions were increased, and LC3B-II expressions were decreased (Figure 5B, *P* < 0.05). Furthermore, compared to the LPS + melatonin group, LPS + melatonin + Compound C treatment increased Bax/Bcl-2 ratio and increased myocardial apoptosis (Figure 5A,B, *P* < 0.05). These results showed that Compound C significantly abolished the protective effects of melatonin, as indicated by an increased inflammation level, decreased autophagy level, increased apoptosis level and decreased cardiac function.

**Figure 6:**
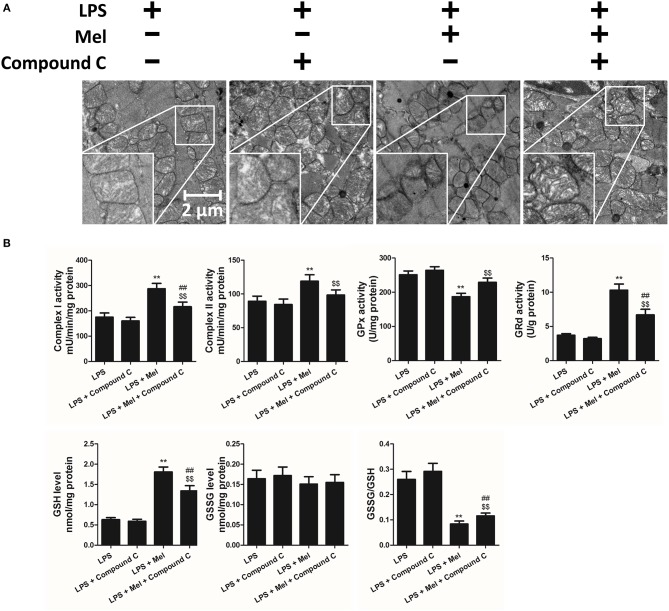
Effects of Compound C treatment on mitochondrial morphology and function. **(A)** Representative images of mitochondrial morphology. **(B)** Quantitative analysis of mitochondrial Complex I, Complex II, GPx, and GPd activity, and GSH, GSSG levels. *n* = 6 **P < 0.05 vs. the LPS group, ^##^*P* < 0.05 vs. the LPS + melatonin group, ^*$$*^*P* < 0.05 vs. the LPS + Compound C group. Mel, melatonin.

TEM was carried out to determine the micro-histopathology of mouse heart tissue. Compared to the LPS group, melatonin decreased LPS-induced injury, as indicated by less swelling mitochondria. Compared to the LPS + melatonin group, Compound C treatment abolished the protective effects of melatonin (Figure 6A). Furthermore, mitochondria function was detected to confirm the morphological changes. Compared to LPS group, LPS + melatonin treatment decreased mitochondria injury by increasing Complex I activity, Complex II activity, GRd activity and GSH levels and by decreasing GPx activity and GSSG levels (Figure 6B, *P* < 0.05). Compared to the LPS + melatonin group, Compound C decreased Complex I activity, Complex II activity, GRd activity and GSH levels and increased GPx activity and GSSG levels (Figure 6B, *P* < 0.05).

### Autophagy Is Regulated by AMPK and Plays a Critical Role in Melatonin Induced Myocardial Protection

**Figure 7:**
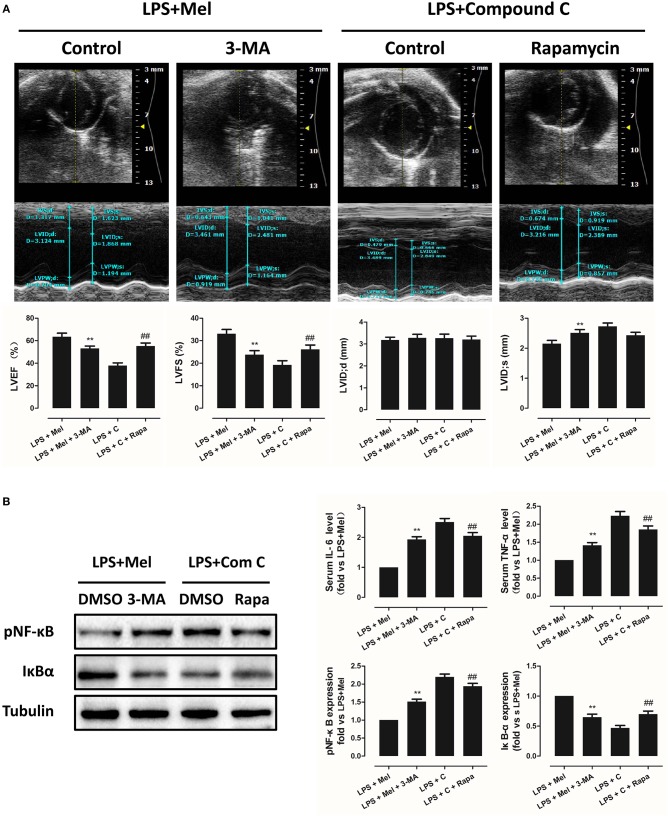
Autophagy inhibition impaired the cardiac protection of melatonin, and autophagy activation rescued AMPK inhibition induced cardiac injury. **(A)** Representative M-mode echocardiograms from mice in each group and quantitative analysis of mice LVEF, FS, LVID; d, LVID; s. **(B)** Representative bands of pNF-κB and IκBα, and quantitative analysis of pNF-κB, IκBα expressions and Serum IL-6, TNF-α levels. *n* = 6. **P < 0.05 vs. the LPS + melatonin group, ^##^*P* < 0.05 vs. the LPS + Compound C group. Mel, melatonin; C, Compound C; Rapa, rapamycin.

**Figure 8:**
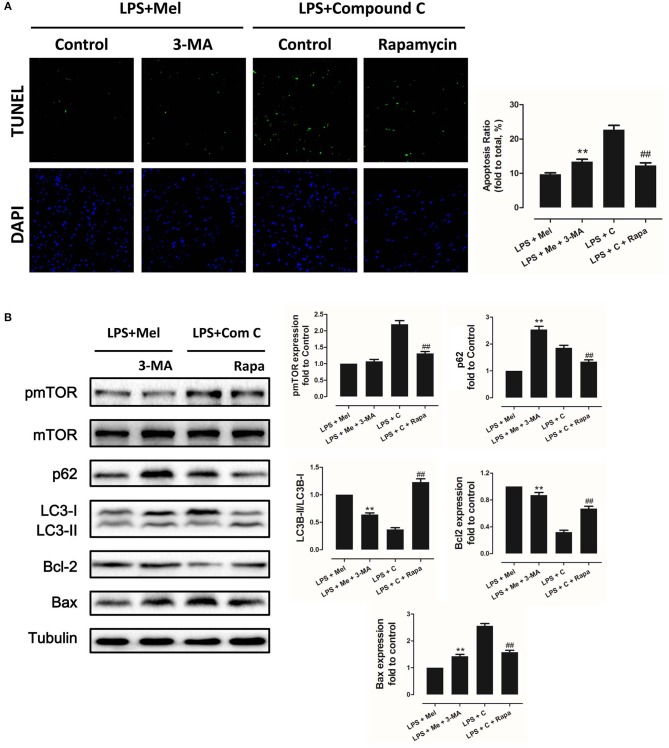
Autophagy inhibition increased myocardial apoptosis, and autophagy activation decreased Compound C induced myocardial apoptosis. **(A)** Representative images of TUNEL staining and quantitative analysis of the apoptosis ratio. **(B)** Representative bands of western blot and quantitative analysis of pmTOR phosphorylation levels, p62 expression, LC3B-II/LC3B-I ratio, Bcl-2 expression and Bax expression. *n* = 6, **P < 0.05 vs. the LPS + melatonin group, ^##^*P* < 0.05 vs. the LPS + Compound C group. Mel, melatonin; C, Compound C; Rapa, rapamycin.

**Figure 9:**
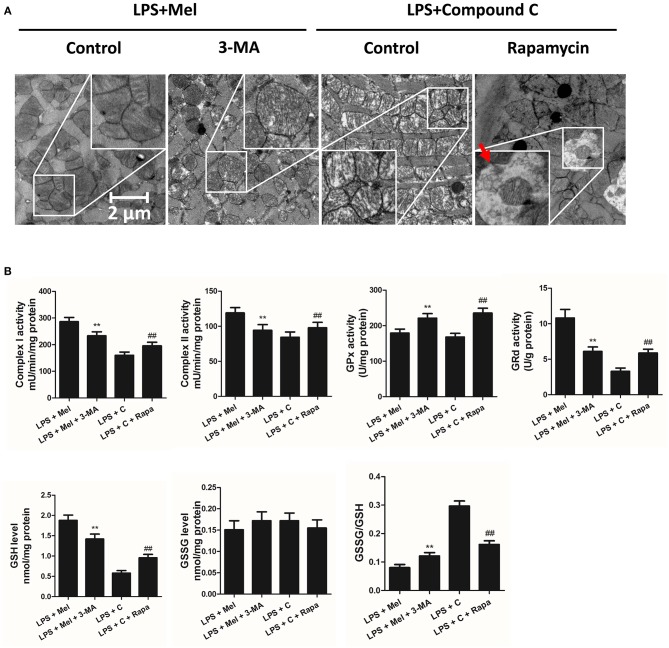
Autophagy inhibition increased mitochondrial function, and autophagy activation decreased Compound C induced mitochondrial dysfunction. **(A)** Representative images of mitochondrial morphology. The red arrow indicates an autophagosome **(B)** Quantitative analysis of mitochondrial Complex I, Complex II, GPx and GPd activity, and GSH, GSSG levels. *n* = 6 **P < 0.05 vs. the LPS + melatonin group, ^##^*P* < 0.05 vs. the LPS + Compound C group. Mel, melatonin; C, Compound C; Rapa, rapamycin.

Then we further validate the key role of autophagy in LPS-induced heart injury. It was shown that melatonin exerts cardiac protection via activating AMPK and autophagy. Effects of autophagy inhibition was investigated by utilizing 3-MA. Compared to the LPS + melatonin group, administration of 3-MA significantly reduced LVEF and LVFS and increased LVID;s (Figure 7A, *P* < 0.05). In addition, 3-MA increased pro-inflammatory cytokines levels of IL-6, TNF-α and pNF-κB and decreased IκBα expression (Figure 7B, *P* < 0.05). 3-MA significantly reduced LC3B-II/LC3B-I ratio and increased Bax and decreased Bcl-2 expression (Figure 8B, *P* < 0.05). Furthermore, 3-MA increased myocardial apoptosis (Figure 8A, *P* < 0.05). TEM detection showed that 3-MA increased mitochondria injury (Figure 9A). 3-MA also decreased Complex I activity, Complex II activity, GRd activity and GSH levels and increased GPx activity and GSSG levels (Figure 9B, *P* < 0.05).

It has been proved that AMPK-regulated autophagy is involved in melatonin induced cardio protection. We further activated autophagy at the presence of Compound C to validate that autophagy is regulated by AMPK in this process. Administration of Rapa rescued LPS + Compound C induced heart injury, as indicated by higher LVEF and LVFS and lower IL-6, TNF-α and pNF-κB levels (Figure 7B, *P* < 0.05). Western blot analysis showed that Rapa activated autophagy via reducing pmTOR and increasing LC3B-II/LC3B-I ratio (Figure 8B, *P* < 0.05). Rapa also decreased Bax/Bcl-2 ration and decreased myocardial apoptosis (Figures 8A,B, *P* < 0.05). TEM detection showed that Rapa treatment decreased mitochondria injury compared to the LPS + Compound C group (Figure 9A). Furthermore, Rapa treatment increased Complex I activity, Complex II activity, GRd activity and GSH levels and decreased GPx activity and GSSG levels (Figure 9B, *P* < 0.05). These results demonstrated the relationship between melatonin, AMPK, autophagy, and apoptosis. Melatonin rescues LPS-induced myocardial injury via activating AMPK/mTOR/autophagy pathway and inhibiting apoptosis.

### Melatonin Reversed LPS-Induced Inflammation and Reduced Apoptosis Through AMPK/mTOR/autophagy Pathway, Which Can Be Abolished by Compound C Treatment *in vitro*

**Figure 10:**
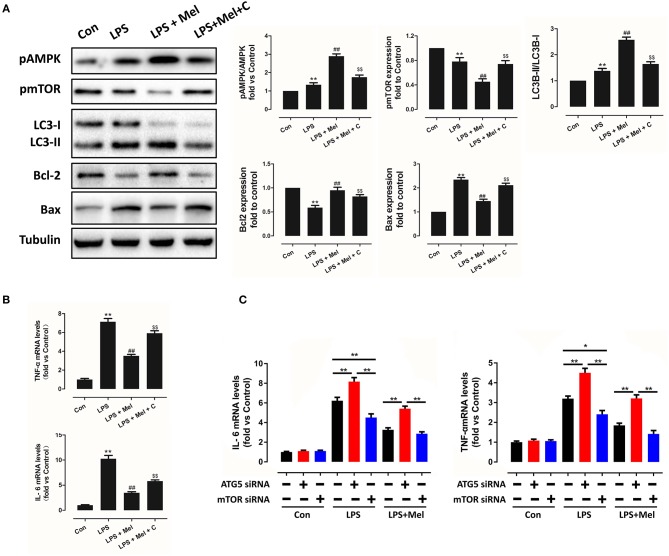
Melatonin reversed LPS-induced inflammation and reduced apoptosis through the AMPK/mTOR/autophagy pathway, which can be abolished by Compound C treatment *in vitro*. **(A)** Representative bands and quantitative analysis of pAMPK phosphorylation levels, pmTOR phosphorylation levels, LC3B-II/ LC3B-I ratio, Bcl-2 expression and Bax expression. **(B)** Cell IL-6 mRNA levels and cell TNF-α mRNA levels. *n* = 6, **P < 0.05 vs. the Control group, ^##^*P* < 0.05 vs. the LPS group, ^*$$*^*P* < 0.05 vs. the LPS + melatonin group. **(C)** Cell IL-6 mRNA levels and cell TNF-α mRNA levels, *n* = 6. *P < 0.05, **P < 0.01.

To further validate this mechanism, *in vitro* experiments were performed on primary neonatal rat cardiomyocytes. Neonatal cardiomyocytes of rats are commonly used in cardiac studies. In previous studies, *in vitro* experiments based on rat cardiomyocytes and *in vivo* experiments based on mice or rats were conducted to prove their hypothesis (48, 49). LPS treatment increased IL-6, TNF-α mRNA levels (Figure 10B, *P* < 0.05), increased Bax and reduced Bcl-2 expression (Figure 10A, *P* < 0.05); melatonin administration reversed this trend. Melatonin also increased LC3B-II/LC3B-I ratio. However, the protection of melatonin was abolished by Compound C, indicating that AMPK mediates protection of melatonin, which is consistent with the *in vivo* study.

In addition, to validate the key role of autophagy in LPS-induced inflammation, siRNAs targeting ATG5 and mTOR were utilized to knockdown key proteins of autophagy. Compared to the LPS group, knocking down ATG5 further increased IL-6 and TNF-α mRNA levels (Figure 10C, *P* < 0.05), while knocking down mTOR reduced IL-6 and TNF-α mRNA levels, compared to the scrambled control siRNA group (Figure 10B, *P* < 0.05), indicating activation of autophagy mediates melatonin induced protection against LPS-induced myocardial injury.

## Discussion

In this study, we found that melatonin exerted myocardial protection against sepsis via AMPK-regulated autophagy activation and mitochondrial function preservation.

As an important characteristic of sepsis, LPS-induced acute myocardial injury is responsible for septic shock and significantly increases mortality rates (6, 8). Melatonin is an endogenously released indoleamine which coordinates circadian rhythms with external light-dark cycle. In addition, studies have confirmed the multiple pharmacological effects of melatonin. Melatonin provides a strong defense against septic myocardial injury. In addition to the protection against cardiovascular disorders (28–33), melatonin is reported to protect against sepsis and septic organ dysfunction (5). To be specific, melatonin significantly alleviates LPS-induced liver mitochondrial dysfunction (50), LPS-induced blood-brain barrier disruption (51), and oxidative stress injury in skeletal muscle (52). In particular, melatonin administration also significantly improves vascular hyporeactivity and circulatory failure in endotoxemic rats (53). Further study showed that melatonin could significantly ameliorate mitochondrial impairment and improve the survival rate in animals with septic myocardial injury (54, 55). However, the underlying mechanism of these protective effects has not been fully understood. In the current study, we observed that melatonin administration significantly increased the survival rate of septic mice. Furthermore, melatonin reversed LPS-induced cardiac dysfunction and improved cardiac function, as evidenced by LVEF, LVFS and LVID; s (Figure 1).

Septic organ dysfunction is closely related with the excessive production of numerous pro-inflammatory cytokines, such as TNF-α, IL-1β, gamma interferon (INF-γ), and IL-6 (5, 9, 10). Melatonin is an accepted anti-inflammatory molecule (22, 23). NF-κB activation in septic kidney injury is found to be suppressed by melatonin (56). Notably, inhibition of melatonin against Nod-like receptor protein 3 (NLRP3) inflammasome activation in the heart during sepsis implied cardioprotective effects of melatonin against sepsis-induced acute myocardial injury (56, 57). In this study, we observed that LPS-induced NF-κB activation in heart tissue and IL-6 and TNF-α elevation in the serum were significantly attenuated by melatonin administration (Figure 1). These results indicated that melatonin may protect against septic myocardial injury through anti-inflammatory mechanisms.

As an important cellular process, autophagy disassembles damaged organelles in cells, which is also reported to participate in the pathogenesis of sepsis (58). A recent study showed that a decreased expression of autophagy protein is associated with increased secretion of inflammatory cytokines and increased severity of sepsis (58). Dampened autophagy significantly contributes to LPS-induced myocardial inflammation and dysfunction (59). In this study, we detected autophagy activation after melatonin treatment (Figures 2B, 3A). Furthermore, we also found that administration of the autophagy inhibitor, 3-MA, significantly increased the serum levels of cytokines, which reversed the anti-inflammatory and cardioprotective effects of melatonin. (Figure 7) The protective effects of autophagy in LPS-induced septic organ dysfunction have also been reported in previous studies (60, 61). In particular, preservation of mitochondrial function and activation of autophagy may be a potential therapeutic opportunity for septic myocardial injury (11, 12, 62, 63). Interestingly, melatonin has been reported as an autophagy regulator (36, 37). In this study, Complex I, Complex II, GPx, GRd activities, GSH levels, as well as mitochondrial ultrastructure were detected to assess mitochondrial function. We observed that administration of melatonin activated autophagy and further exerted cardioprotective effects against LPS-induced mitochondrial and myocardial dysfunction, which could be impaired by 3-MA (Figures 1–3, 7–9).

AMPK is a key enzyme in maintaining energy homeostasis (64). As a cellular energy sensor, AMPK regulates several cellular processes, including autophagy (65). As an important controller of cell fate, mTOR acts as a downstream effector of AMPK (65, 66). To be specific, activation of AMPK induces the formation of the AMPK/mTOR complex, which down-regulates phosphorylated mTOR levels and promotes autophagic flux. Activation of AMPK-mediated autophagy flux is found to protect against septic liver and kidney injury (67, 68). In particular, during septic myocardial dysfunction, AMPK activation promotes autophagy, which protects against LPS-induced cardiotoxicity (59, 63). In our study, we found that AMPK activation and phosphorylation significantly attenuated mitochondrial damage and myocardial dysfunction by decreasing pmTOR and p62 expressions and activating autophagy (Figures 2–3), which could be inhibited by Compound C (Figures 4–6). Furthermore, the protective effects of AMPK activation were further abolished by the autophagy inhibitor 3-MA (Figures 7–9), indicating the important roles of AMPK-mediated autophagy in this process. Interestingly, AMPK inhibition by Compound C exacerbated LPS induced heart injury, indicated by worse myocardial function, higher apoptosis levels and more sever mitochondria damage. Considering that activation of AMPK can be observed in LPS group, AMPK moderate activation might be an adaptive response to LPS insult. In consistent with our finding, Sun and colleagues also discovered that LPS insult mildly activated autophagy (69). AMPK activation may represent an adaptive event to stress or exercise to maintain cardiac energy homeostasis (70). Compound C abolished this response and leads to a worse condition. Another interesting phenomenon is that AMPK inhibition or autophagy inhibition did not totally abolish melatonin exerted myocardial protection, which means AMPK regulated autophagy is not the only mechanism. Melatonin might regulate multiple pathways to attenuate LPS induced myocardial injury, and cross-talks between these pathways still need to be investigated.

Our previous studies showed that AMPK is an important target of melatonin (10, 71). Melatonin is found to exert protective effects via AMPK activation-mediated autophagic flux (72, 73). AMPK is activated and phosphorylated during the protection of melatonin against septic organ dysfunction, such as blood brain barrier disruption (51, 74). In this study, we found that melatonin is cardioprotective against septic myocardial dysfunction; the mechanism of melatonin involved consisted of the activation of AMPK and autophagy. Furthermore, melatonin also inhibited the inflammatory response, mitochondrial damage and apoptosis of myocardial cells.

**Figure 11:**
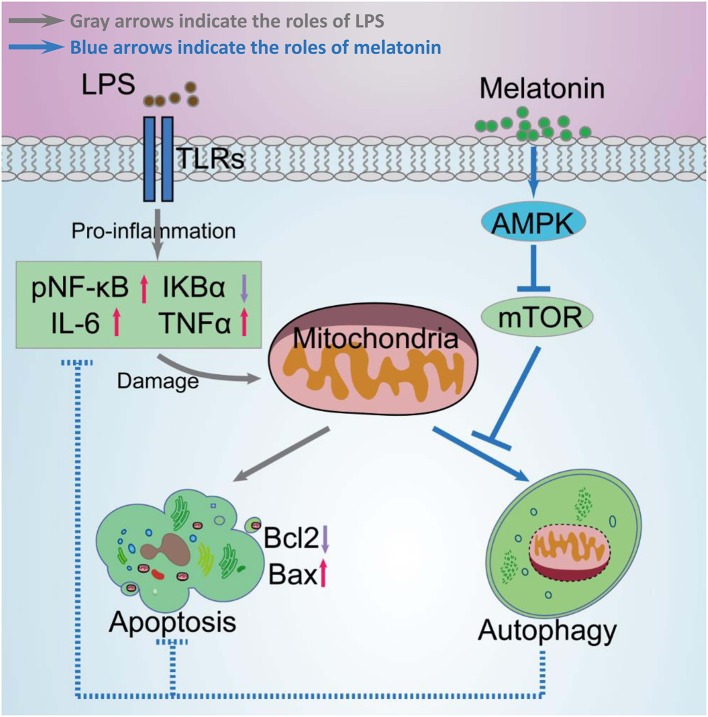
Schematic diagram of melatonin induced protection against LPS-induced acute myocardial injury primarily via AMPK/mTOR/autophagy signaling. In this schematic diagram, gray arrows indicate the effects of LPS and blue arrows indicate the effects of melatonin. LPS significantly increases pNF-κB, IL-6, TNF-α levels, and decreases IκBα levels, which further damages mitochondrial function and increases apoptosis. Melatonin activated AMPK and further activated autophagy through suppressing mTOR. Activation of autophagy protected cell from mitochondria damage related apoptosis and decreased inflammation levels.

Taken together, melatonin attenuated LPS-induced myocardial dysfunction and apoptosis through activating AMPK and autophagy, and further inhibiting inflammatory response and preserving mitochondrial function. Melatonin is a promising reagent for prevention of sepsis, but further studies are still need before clinical application of melatonin (Figure 11).

## Data Availability Statement

The raw data supporting the conclusions of this article will be made available by the authors, without undue reservation, to any qualified researcher.

## Ethics Statement

This study was carried out in accordance with the recommendations of the National Institutes of Health Guidelines for the Use of Laboratory Animals with written informed consent from all subjects. All subjects gave written informed consent in accordance with the Declaration of Helsinki. The protocol was approved by the Fourth Military Medical University Committee on Animal Care.

## Author Contributions

JG and XL conceived and designed the research. SD, ZW, and WH performed the experiments. SD, ZW, WH, XY, WL, and ZM analyzed the data, prepared the figures, and drafted the manuscript. SD, ZW, and WH interpreted results of the experiments.

### Conflict of Interest

The authors declare that the research was conducted in the absence of any commercial or financial relationships that could be construed as a potential conflict of interest.
